# Exercise, energy balance and body composition

**DOI:** 10.1038/s41430-018-0180-4

**Published:** 2018-09-05

**Authors:** Klaas R Westerterp

**Affiliations:** NUTRIM, Maastricht University Medical Centre, Maastricht, The Netherlands

## Abstract

Activity-induced energy expenditure, as determined by the activity pattern including exercise, is the most variable component of daily energy expenditure. Here, the focus is on effects of exercise training on energy balance and body composition in subjects with a sedentary or light-active lifestyle. Then, exercise training induces an energy imbalance consistently lower than prescribed energy expenditure from exercise. Additionally, individual responses are highly variable and decrease in time. Combining the results from 23 exercise training studies in normal-weight, overweight, and obese subjects, varying in duration from 2 to 64 weeks, showed an average initial energy imbalance of about 2 MJ/day with an exponential decline to nearly zero after about 1 year. A compensatory increase in energy intake is the most likely explanation for the lower than expected effect of exercise on energy balance. Overall, exercise training results in a healthier body composition as reflected by a reduction of body fat, especially in overweight and obese subjects, with little or no long-term effect on body weight.

## Basics in clinical nutrition

Activity-induced energy expenditure is the most variable component of daily energy expenditure (DEE), as determined by the activity pattern including exercise. Variation in energy expenditure determines, with energy intake, energy balance, and eventually body composition, when energy imbalance is covered by storage or mobilization of body fat. Additionally, consistent changes in physical activity through immobilization or exercise training affect body composition by changes in muscle mass. Thus, elite athletes like participants in Olympic events have a lower body fat percentage than the average value for similar aged subjects of the general population [1]. Body mass index in athletes is a better proxy for muscle mass than for adiposity [2].

The elite athlete is an example of maximum sustained energy expenditure while maintaining energy balance [3]. Endurance athletes, like Tour de France participants consume energy-dense carbohydrate-rich foods and liquid formulas in order to compete at top level [4]. In the general population, physical activity level (PAL), calculated as DEE divided by resting energy expenditure (REE, PAL = DEE/REE) reaches a maximum value of 2.00–2.40 as shown by well-controlled exercise intervention studies [5]. FAO/WHO/United Nations University classified PAL into three categories: 1.40–1.69 for a sedentary or light-active lifestyle; 1.70–1.99 for active or moderately active lifestyles; and 2.00–2.40 for a vigorously active lifestyle [6]. Here, the focus is on effects of exercise on energy balance and body composition in subjects with light to moderately active lifestyle, that is, an initial PAL around the population average of 1.75.

Studies of exercise affecting energy balance and body composition necessarily involve observations over weeks rather than days. In military cadets with day-to-day changes in exercise expenditure, energy balance varied from day-to-day as well, but energy intake correlated with expenditure over weeks, and even better over longer intervals [7]. Additionally, energy imbalances have to be large or to be sustained over longer time to result in detectable changes in body composition. A change in fat mass that can be measured with a three-compartment model for body composition in an individual subject has to be larger than 1.5 kg, equivalent to about 60 MJ [8]. Exercise energy expenditure of a normal-weight adult with a PAL of 1.75 is 3–4 MJ per day and 20–30 MJ per week [6]. Thus, the minimum intervention interval of exercise studies included in the analysis was 2 weeks.

Thomas et al. [9] evaluated effects of exercise on energy balance, based on data published until 2012. The analysis covered 15 studies with a total of 657 subjects, 518 women, and 139 men, age 21–69 years, body mass index 20–35 kg/m^2^, and intervention interval 3–64 weeks. In two three-week studies, under fully controlled confined conditions, an exercise-induced negative energy balance was equivalent to the calculated change in body energy stores. For the majority of studies, being longer and under less controlled conditions, the achieved energy imbalance was consistently lower than prescribed energy expenditure from exercise. One explanation is compliance with an exercise program without compensating for an exercise-induced increase in energy expenditure by increasing energy intake. Intake compensation was suggested to be a function of baseline body composition. Studies under confined conditions suggested a compensatory increase in intake to an exercise-induced increase in expenditure might not begin until body energy stores are depleted [10, 11].

## Present research activities

In normal-weight subjects, exercise training had little or no effect on body weight [12]. A long-term intervention study, training sedentary women and men with a body mass index between 19 and 26 kg/m^2^ to run a half-marathon after 44 weeks, showed no change in body weight until the final observation at 40 weeks. Then, men showed a median weight loss of 1 kg and similar losses in women were not significant. However, there were pronounced changes in body composition, with a loss in fat mass nearly fully compensated by an increase in fat-free mass. In men, the loss in fat mass was positively correlated with initial percentage body fat. Thus, exercise training resulted in a healthier body composition, especially for subjects with larger body fat stores.

In overweight and obese subjects, exercise training resulted in effects on body weight and body composition with large inter-individual variation. Sedentary overweight and obese women and men, subjected to a 12-week intervention with five supervised exercise sessions per week, showed individual changes ranging from −14.7 to +1.7 kg for body weight and from –9.5 to +2.6 kg for body fat [13]. Mean weight loss of 3.7 ± 3.6 kg was comparable to mean fat loss of 3.7 ± 2.6 kg. The energy content of body fat loss was close to the programmed exercise expenditure of 2.1 MJ (500 kcal) per session, resulting in 12 × 5 × 2.1 = 126 MJ for the total intervention, equivalent to 3.2 kg fat. A 10-month exercise intervention in overweight and obese women and men, also with five supervised exercise sessions per week, resulted in similar inter-individual variation [14]. A group exercising at 1.7 MJ (400 kcal) per session lost 3.9 ± 4.9 kg and a group exercising at 2.5 MJ (600 kcal) per session lost 5.2 ± 5.9 kg body weight and again, mean values for fat loss were comparable to weight loss. However, fat loss over 10 months was not much larger than fat loss over the similar 3-month intervention of the earlier study described above. Thus, individual responses of exercise training on energy balance and body composition in overweight and obese subjects are highly variable and reach a plateau in time.

So far, an evidence-based explanation for the large variation in response of exercise training on energy balance and body composition is lacking. Potential explanations are an effect of exercise training on energy intake, non-exercise physical activity, and/or REE. Energy intake measurement for the assessment of energy balance, as based on self-report, is not sufficiently accurate [15]. In normal-weight subjects training to run a half-marathon after 44 weeks [16], non-REE increased 2.5 MJ per day while self-reported intake remained unchanged, suggesting a cumulative energy imbalance equivalent to 20 kg fat, five to ten times more than the observed body fat loss [5, 12]. Reviews on behavioral changes in response to exercise training showed no clinical significant changes in non-exercise physical activity during the initiation and adaptation to exercise [17–19]. A review of studies on exercise effects on energy balance in sedentary subjects could only conclude that REE does not change as long as body weight is maintained [9].

## Need of future research

Knowledge about mechanisms behind variation in responses of exercise training on energy balance and body composition is useful to optimize exercise for prevention and treatment of overweight and obesity. The largest part of the response variability is ascribed to exercise-induced changes in energy intake [20]. An answer might come from a large randomized controlled exercise study in sedentary overweight and obese subjects, completed in 2015 (ClinicalTrials.gov ID: NCT01264406) [21]. In this study, energy intake will be derived from energy expenditure measured with doubly labeled water, adjusted for changes in body composition. Additional questions to be answered are: what limits exercise-induced fat loss; why does an exercise-induced change in body composition plateau in time; and is exercise intensity and exercise volume critical to reduce fat mass.

Exercise-induced fat loss is limited by the maximum sustained PAL. The doubly labeled water assessed PAL of overweight subjects and obese is similar to the PAL of normal-weight subjects with a mean value around 1.75 [5]. Activity-induced energy expenditure is similar or slightly higher in most obese subjects. Obese subjects can move less for the same amount of energy through the increased cost of moving a larger body mass [22]. High body weight leads to high activity-induced energy expenditure, even when moving less than normal-weight subjects [23]. With exercise training, resulting in an additional energy expenditure of 2–3 MJ per day, the PAL reaches its maximum value of 2.0–2.4. Thus, the maximum exercise-induced fat loss is about 0.5 kg per week.

**Fig. 1:**
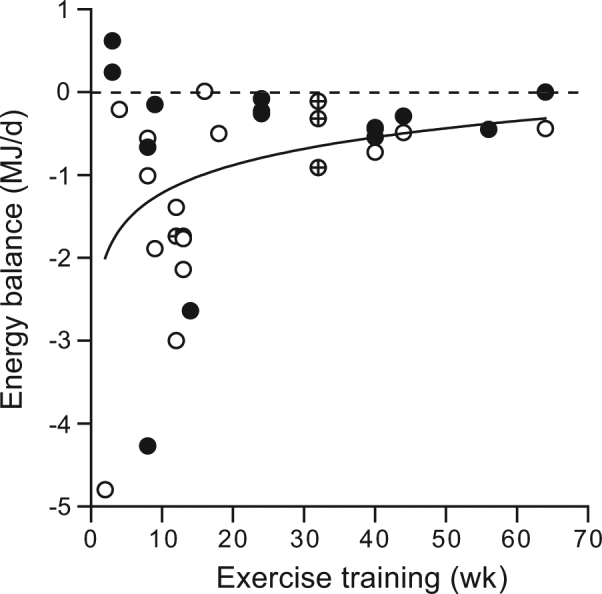
Energy balance for studies with different duration of exercise training as presented in Table 1. Energy balance is calculated from the change in body composition using the chemical energy equivalents for changes in fat mass (39.5 MJ/kg) and fat-free mass (7.6 MJ/kg) [32]. Closed dots: women; open dots: men; and crossed dots: women and men

An exercise-induced negative energy balance decreases in time, as shown by a compilation of data from 23 exercise training studies varying in duration from 2 to 64 weeks (Table 1 and Fig. 1). The shortest study was in men reaching an extremely high PAL value of about four, during a cycling race over 2706 km, resulting in the most negative energy balance of 4.8 MJ per day, without an effect on body weight [24]. Fat loss, to cover the energy deficit, was similar to the exercise-induced gain in fat-free mass. The longest study was in overweight women and men performing 64 weeks supervised walking and biking exercise increasing to 1.2 MJ per day, resulting in a weight loss in men and no weight change in women [25]. Men lost 5.2 kg weight, mainly as fat, and women showed no exercise-induced change in body composition. Most studies, subjecting women and men to the same exercise intervention, report no or non-significant gender differences for exercise-induced changes in body composition [13, 14, 26–29].

**Table 1:** Exercise training studies with intervention length and observed average changes in body weight and body fat

Reference	Subjects women/men	Exercise	Interval (week)	∆ Weight (kg)	∆ Body fat (kg)
[10]	3/0	Treadmill 2.5 MJ per day	8	−6.8	−6.0
[11]	5/0	Treadmill 1.6 and 3.2 MJ per day	3	0.5^NS^ and 0.1	0.3^NS^ and 0.2^NS^
[33]	0/4	Treadmill 2.1 MJ per day	12	−5.1	−3.3
[26]	3/3	Jogging 20 to 60 min per day	9	−0.9	−1.8^NS^
[34]	13/0	Cycling 1.5 MJ per day	56	−3.7	−4.6
[12]	11/12	Jogging 20 to 40 min per day	44	−1.0	−3.5
[35]	0/10	Cycling 0.9 MJ per day	4	0.5^NS^	−0.3^NS^
[36]	5/6	Cycling 0.6 MJ per day	8	0.0	−0.9
[37]	0/14	Cycling 2.6 MJ per day	13	−5.0	−4.9
[38]	0/18	Resistance 0.6 MJ per day	18	0.1^NS^	−2.0
[39]	0/16	Treadmill 2.9 MJ per day	12	−7.5	−6.1
[25]	25/16	Treadmill 1.2 MJ per day	64	−1.7	−2.1
[40]	17/0	Treadmill 2.1 MJ per day	14	−5.9	−6.7
[27]	37/46	Treadmill 0.6 and 1.4 MJ per day	32	−1.1 and −3.5	−2.0 and −4.9
[13]	25/10	Treadmill/cycling 1.5 MJ per day	12	−3.7	−3.7
[41]	309/0	Treadmill/cycling 0.2 and 0.5 MJ per day	24	−1.4 and −2.1	−0.1^NS^ and −0.7^NS^
[28]	52/14	Treadmill/cycling 0.9 MJ per day	32	−0.6 ^ns^	−0.6
[42]	0/36	Jogging/cycling 1.25 and 2.5 MJ per day	13	−3.6 and −2.7	−4.0 and −3.8
[14]	37/37	Treadmill 1.2 and 1.8 MJ per day	40	−3.9 and −5.2	−3.5 and −5.2
[24]	0/6	Cycling 193 km per day	2	0.4^NS^	−2.2
[43]	0/9	Treadmill/resistance 0.4 to 0.8 MJ per day	16	0.5^NS^	−0.1^NS^
[44]	0/46	Jogging/cycling 1.5 MJ per day	8	−1.4	−1.6
[29]	41/38	Jogging/cycling 0.9 MJ per day	24	−1.1	−1.1

Compensatory changes explain the decrease of an exercise-induced disturbance of energy balance in time [30]. In addition to compensatory changes in energy intake, DEE does not increase linearly with increasing exercise volume. A cross-sectional analysis showed sedentary individuals tend to adapt metabolically to increased physical activity [31]. A longitudinal study showed an exercise-induced increase in activity-induced energy expenditure, to reach a plateau despite a further doubling of the exercise volume [5]. Training increases exercise economy, especially in sedentary untrained subjects.

In conclusion, in sedentary subjects exercise does affect energy balance and body composition. The achieved energy imbalance is generally lower than prescribed energy expenditure from exercise, especially in normal-weight subjects. In overweight and obese subjects, individual responses of exercise training on energy balance and body composition are highly variable, and reach a plateau in time. At a group level, exercise training results in negative energy balance of about 2.0 MJ per day with an exponential decline to 0.5–0.0 MJ per day in studies longer than 1 year. Exercise training effects on REE and non-training activity are negligible. The most likely explanation for a return to energy balance is a compensatory increase in energy intake.

## References

[1] Fleck SJ (1983). Body composition of American athletes. Am J Sports Med.

[2] Nevill AM, Winter EM, Ingham SA, Watts AS, Metsios G, Stewart AD (2010). Adjusting athletes’ body mass index to better reflect adiposity in epidemiological research. J Sports Sci.

[3] Cooper JA, Nguyen DD, Ruby BC, Schoeller DA (2011). Maximal sustained levels of energy expenditure in humans during exercise. Med Sci Sports Exerc.

[4] Westerterp KR, Saris WHM (1991). Limits of energy turnover in relation to physical performance, achievement of energy balance on a daily basis. J Sports Sci.

[5] Westerterp KR (2018). Exercise, energy expenditure and energy balance, as measured with doubly labelled water. Proc Nutr Soc.

[6] FAO/WHO/UNU. Human energy requirements. FAO Food and Nutrition Technical Report Series no. 1. Rome: Joint FAO/WHO/UNU Expert Consultation. 2004.

[7] Edholm OG, Fletcher JG, Widdwson EM, McCance RA (1955). The energy expenditure and food intake of individual men. Br J Nutr.

[8] Jebb SA, Murgatroyd PR, Goldberg GR, Prentice AM, Coward WA (1993). In vivo measurement of changes in body composition: description of methods and their validation against 12-d continuous whole-body calorimetry. Am J Clin Nutr.

[9] Thomas DM, Bouchard C, Church T, Slentz C, Kraus WE, Redman LM (2012). Why do individuals not lose more weight from an exercise intervention at a defined dose? An energy balance analysis. Obes Rev.

[10] Woo R, Garrow JS, Pi-Sunyer FX (1982). Voluntary food intake during prolonged exercise in obese women. Am J Clin Nutr.

[11] Woo R, Pi-Sunyer X (1985). Effect of increased physical activity on voluntary intake in lean women. Metabolism.

[12] Westerterp KR, Meijer GAL, Janssen EME, Saris WHM, Ten Hoor F (1992). Long-term effect of physical activity on energy balance and body composition. Br J Nutr.

[13] King NA, Hopkins M, Caudwell P, Stubbs RJ, Blundell JE (2008). Individual variability following 12 weeks of supervised exercise: identification and characterization of compensation for exercise-induced weight loss. Int J Obes.

[14] Donnelly JE, Honas JJ, Smith BK, Mayo MS, Gibson CA, Sullivan DK (2013). Aerobic exercise alone results in clinically significant weight loss for men and women: Midwest exercise trial-2. Obesity.

[15] Dhurandhar NV, Schoeller D, Brown AW, Heymsfield SB, Thomas D, Sørensen TIA (2015). Energy balance measurement: when something is not better than nothing. Int J Obes.

[16] Westerterp KR (1998). Alterations in energy balance with exercise. Am J Clin Nutr.

[17] Melanson EL, Keadle SK, Donnelly JE, Braun B, King NA (2013). Resistance to exercise-induced weight loss: compensatory behavioral adaptations. Med Sci Sports Exerc.

[18] Washburn RA, Lambourne K, Szabo AN, Herrmann SD, Honas JJ, Donelly JE (2014). Does increased prescribed exercise alter non-exercise physical activity/energy expenditure in healthy adults? A systematic review. Clin Obes.

[19] Fedewa MV, Hathaway ED, Williams TD, Schmidt MD (2017). Effect of exercise training on non-exercise physical activity: a systematic review and meta-analysis of randomized controlled trials. Sports Med.

[20] Blundell JE, Gibbons C, Caudwell P, Finlayson G, Hopkins M (2015). Appetite control and energy balance: impact of exercise. Ob Rev.

[21] Myers CA, Johnson WD, Earnest CP, Rood JC, Tudor-Locke C, Johannsen NM (2014). Examination of mechanisms (E-MECHANIC) of exercise-induced weight compensation: study protocol for a randomized controlled trial. Trials.

[22] Ekelund U, Åman J, Yngve A, Renman C, Westerterp K, Sjöström M (2002). Physical activity but not energy expenditure is reduced in obese adolescents: a case–control study. Am J Clin Nutr.

[23] DeLany JP, Kelly DE, Hames KC, Kakicic JM, Goodpaster BH (2013). High energy expenditure masks low physical activity in obesity. Int J Obes.

[24] Rosenkilde M, Morville T, Andersen PR, Kjaer K, Rasmussen H, Holst JJ (2015). Inability to match energy intake with energy expenditure at sustained near-maximal rates of energy expenditure in older men during a 14-d cycling expedition. Am J Clin Nutr.

[25] Donnelly JE, Hill JO, Jacobsen DJ, Potteiger J, Sullivan DK, Johnson SL (2003). Effect of 16-month randomized controlled exercise trial on body weight and composition in young, overweight men and women. Arch Intern Med.

[26] Bingham SA, Goldberg GR, Coward WA, Prentice AM, Cummings JH (1989). The effect of exercise and improved physical fitness on basal metabolic rate. Br J Nutr.

[27] Slentz CA, Duscha BD, Johnson JL, Ketchum K, Aiken LB, Samsa GP (2004). Effects of the amount of exercise on body weight, body composition, and measures of central obesity: STRRIDE—a randomized controlled study. Arch Intern Med.

[28] Church TS, Earnest CP, Thompson AM, Priest EL, Rodarte RQ, Saunders T (2010). Exercise without weight loss does not reduce C-reactive protein: the INFLAME study. Med Sci Sports Exerc.

[29] Quist JS, Rosenkilde M, Petersen MB, Gram AS, Sjödin A, Stallknecht B (2018). Effects of active commuting and leisure-time exercise on fat loss in women and men with overweight and obesity: a randomized controlled trial. Int J Obes.

[30] Dhurandhar EJ, Kaiser KA, Dawson JA, Alcorn AS, Keating KD, Allison DB (2015). Predicting adult weight change in the real world: a systematic review and meta-analysis accounting for compensatory changes in energy intake or expenditure. Int J Obes.

[31] Pontzer H, Durazo-Arvizu R, Dugas L, Plange-Rhule J, Bovet P, Forrester TE (2016). Contrained total energy expenditure and metabolic adaptation to physical activity in adult humans. Curr Biol.

[32] Hall KD (2008). What is the required energy deficit per unit weight loss?. Int J Obes.

[33] Sopko G, Leon AS, Jacobs DR, Foster N, Moy J, Kuba K (1985). The effects of exercise and weight loss on plasma lipids in young obese men. Metabolism.

[34] Després JP, Poliot MC, Moorjani S, Nadeau A, Tremblay A, Lupien PJ (1991). Loss of abdominal fat and netabolic response to exercise training in obese women. Am J Physiol.

[35] Blaak EE, Westerterp KR, Bar-Or O, Wouters LJ, Saris WH (1992). Total energy expenditure and spontaneous activity in relation to training in obese boys. Am J Clin Nutr.

[36] Goran MI, Poehlman ET (1992). Endurance training does not enhance total energy expenditure in healthy elderly persons. Am J Physiol.

[37] Bouchard C, Tremblay A, Després JP, Thériault, Nadeau A, Lupien PJ (1994). The reponse to exercise with constant energy intake in identical twins. Obes Res.

[38] Van Etten LM, Westerterp KR, Verstappen FT, Boon BJ, Saris WH (1997). Effect of an 18-wk weight-training program on energy expenditure and physical activity. J Appl Physiol.

[39] Ross R, Dagnone D, Jones PJ, Smith H, Paddags A, Hudson R (2000). Reduction in obesity and related comorbid conditions after diet-induced weight loss or exercise-induced weight loss in men. A randomized, controlled trial. Ann Intern Med.

[40] Ross R, Janssen I, Dawson J, Kungl AM, Kuk JL, Wong SL (2004). Exercise-induced reduction in obesity and insulin resistance in women: a randomized controlled trial. Obes Res.

[41] Church TS, Martin CK, Thompson AM, Earnest CP, Mikus CR, Blair SN (2009). Changes in weight, waist circumference and compensatory responses with different doses of exercise among sedentary, overweight postmenopausal women. PLoS ONE.

[42] Rosenkilde M, Auerbach P, Reichkendler MH, Ploug T, Stallknecht M, Sjödin A (2012). Body fat loss and compensatory mechanisms in response to different doses of aerobic exercise—a randomized controlled trial in overweight sedentary males. Am J Physiol.

[43] Drenowatz C, Grieve GL, DeMello MM (2015). Change in energy expenditure and physical activity in response to aerobic and resistance exercise programs. SpingerPlus.

[44] Melzer K, Renaud A, Zurbuchen S, Tschopp C, Lehmann J, Malatesta D (2016). Alterations in energy balance from exercise intervention with ad libitum food intake. J Nutr Sci.

